# Sialic acid as the potential link between lipid metabolism and inflammation in the pathogenesis of atherosclerosis

**DOI:** 10.1590/1414-431X2023e12972

**Published:** 2023-12-11

**Authors:** A.V. Poznyak, D.A. Kashirskikh, A.Y. Postnov, M.A. Popov, V.N. Sukhorukov, A.N. Orekhov

**Affiliations:** 1 Institute for Atherosclerosis Research, Moscow, Russia; 2 Laboratory of Cellular and Molecular Pathology of Cardiovascular System, Federal State Budgetary Scientific Institution, Petrovsky National Research Centre of Surgery (FSBSI “Petrovsky NRCS”), Moscow, Russia; 3 Department of Cardiac Surgery, Moscow Regional Research and Clinical Institute (MONIKI), Moscow, Russia

**Keywords:** Desialylation, Sialic acid, LDL, Atherosclerosis

## Abstract

In the modern world, cardiovascular diseases have a special place among the most common causes of death. Naturally, this widespread problem cannot escape the attention of scientists and researchers. One of the main conditions preceding the development of fatal cardiovascular diseases is atherosclerosis. Despite extensive research into its pathogenesis and possible prevention and treatment strategies, many gaps remain in our understanding of this disease. For example, the concept of multiple low-density lipoprotein modifications was recently stated, in which desialylation is of special importance. Apart from this, sialic acids are known to be important contributors to processes such as endothelial dysfunction and inflammation, which in turn are major components of atherogenesis. In this review, we have collected information on sialic acid metabolism, analyzed various aspects of its implication in atherosclerosis at different stages, and provided an overview of the role of particular groups of enzymes responsible for sialic acid metabolism in the context of atherosclerosis.

## Introduction

Atherosclerosis is a chronic cardiovascular disorder with complex pathogenesis. It leads to severe complications, which in turn often cause morbidity and mortality. Despite the growing understanding of the pathogenesis of atherosclerosis, there are still numerous gaps.

Sialic acid is a derivative of neuraminic acid and has a nine-atom carbon chain. Sialic acids are responsible for maintaining many aspects of cell functioning under normal conditions. Also, sialic acids can define the susceptibility of vascular endothelium to atherogenic plaque formation. Moreover, the level of sialylation is an important factor specifying the properties of circulating molecules, such as low-density lipoprotein (LDL).

More than 50 different forms of sialic acid are known, among which N-glycolylneuramic acid (Neu5Gc), N-acetylneuramic acid (Neu5Ac), deaminoneuramic acid, and their single or multiple O-acetyl derivatives can be named (1). Neu5Gc is not formed in human organisms. Since the human body cannot convert Neu5Ac into Neu5Gc, it cannot synthesize endogenous Neu5Gc. The main source of sialic acid for humans is food intake. The richest sources of sialic acids are milk products and especially red meat. In all other mammals, this reaction is catalyzed by the enzyme cytidine monophosphate N-acetyl-D-hydroxylase neuraminic acid (CMAH) (2). In humans, an inactivating mutation of the CMAH gene causes aberrant expression of a non-functional enzyme. As a result of recent studies, it has been established that the normal metabolic incorporation of non-human Neu5Gc from food sources into human tissues can induce circulating antibodies against Neu5Gc. Later, this can cause the development of chronic inflammation and, in particular, atherosclerosis (3).

Sialic acids and the enzymes that metabolize them play an important role in the development of atherosclerosis. A relationship was established between an increase in cholesterol accumulation in macrophages and smooth muscle cells of the human aorta (which were isolated from the blood of patients with coronary artery disease (CAD) or obtained by treatment with bacterial sialidase) and low content of sialic acids in LDL. Since the accumulation of cholesterol esters in the intima of the arteries is one of the earliest manifestations of atherosclerosis, its development may be facilitated by a change in the level of sialic acids in LDL (4). In addition, it was found that the atherogenic modification of LDL is the desialylation of LDL *in vivo*, carried out by human blood trans-sialidase.

In this review, we summarized data on the various sialic acid-related components of atherosclerosis. Unlike other authors, we analyzed the impact on atherosclerosis of both enzymes responsible for sialic acid metabolism and the lipid modifications caused by sialic acids.

## Sialic acid metabolism

The metabolism of sialic acid is a rather complex process. Neu5Ac is synthesized in the cytoplasm of eukaryotic cells and then transferred to the nucleus, where cytosine-5'-monophosphate (CMP)-Neu5Ac is synthesized. It then passes into the Golgi apparatus for the formation of glycoconjugates via sialyltransferases, which are subsequently secreted or delivered to the cell surface (5).

Sialic acid is a monosaccharide that completes glycan structures. Due to its terminal position and properties, sialic acid can participate in the overall immune response and many cellular events. Due to its resistance to proteolytic cleavage and the provision of negative charges, sialic acid stabilizes the conformation of molecules that are responsible for the normal physiological function of cells (6). It is worth noting that sialic acid functions as a recognizable molecular pattern or as a biological mask. In the second case, sialic acid behaves as an agent that prevents recognition. This is due to the masking of recognition sites (proteins on cell membranes, including receptor molecules or a polysaccharide with a glycan chain). In this way, sialic acid can help protect host cells from pathogen recognition. This makes the cells become “independent”, and thus immunoreactivity is weakened (7). At the same time, sialic acid plays the role of a ligand in various molecules (antibodies, hormones, viruses, bacteria, lectins, etc.). Another important property of sialic acid is that it can promote adhesion, signal transmission, and cell recognition. For example, it has recently been established that in the process of immune regulation, the Sia (sialic acid)-Siglecs (sialic acid-binding immunoglobulin-like lectin) axis participates in important phenomena of molecular and cellular interactions.

Three groups of enzymes are responsible for sialic acid removal. Trans-sialidases (TS) can transfer sialic acid from the end of the glycoconjugate to the new glycoconjugate, sialyltransferases (ST) participate in the biosynthesis of sialoglycolipids and sialoglycoproteins, and sialidases (NEU) can hydrolyze sialic acid from the end of the glycoconjugate (8).

Four isoforms of sialidases were found in human cells: NEU1, NEU2 (cytosolic sialidase), NEU3 (plasma membrane-bound sialidase), and NEU4 (lysosomal or mitochondrial membrane-bound sialidase).

ST are present in the Golgi apparatus as well as in the endoplasmic reticulum. It has also been found that ST can transfer sialic acid from CMP-Neu5Ac to the end chains of glycoprotein and glycolipid. Based on the type of glycoside bond, four classes of ST can be distinguished; these are ST3Gal IV-I, ST6Gal I-II, ST6GalNAc I-VI, and ST8 sialic acid I-VI. Research results indicate that due to various mechanisms, ST can play a dual role in the process of atherosclerosis (9,10).

TS were initially found in trypanosomes. They were also found to be present in lipoprotein subcomponents and human serum. It was determined that for optimal TS activity, there are only three pH levels (3.0, 5.0, and 7.0). At the same time, it is worth noting that TS can be activated even at a neutral pH. Also, when the internal pH level decreases to 5.0 due to macrophages, this is reflected in inflammatory lesions (11).

Sialic acids are usually found at the end of the oligosaccharide chain of glycolipids and glycoproteins. They are quite widely spread in various tissues and fluids of the human body. Speaking about the presence of sialic acid in plasma, we can state its high content in haptoglobin, orosomucoid, fibrinogen, ceruloplasmin, and glucose. Also, a large amount of sialic acid is present in the glycoproteins of leukocytes, erythrocytes, and platelets. It is worth noting that a high level of sialic acid is found on the surface of vascular endothelial cells (12). Sialic acids are a group of negatively charged amino sugars. They are covalently bound to glycoproteins and lipids (in the extracellular matrix, plasma, and glycocalyx). It can also be said that for cellular receptors, the terminal residues of sialic acid can act as ligands. They also have a significant effect on the regulation of apolipoprotein retention in the bloodstream. Cleavage of terminal sialic acid changes the charge of the glycan. It can also cause conformational changes that prevent the interaction of the cellular receptor and glycan (13). Thanks to them, other biological effects of the desialylated molecule can also be transformed (such as in the prevention of proteolytic degradation of glycoproteins, binding of calcium ions, intercellular adhesion, inflammation, etc.). That is why it can be argued that desialylation plays a decisive role in the pathogenesis of atherosclerosis and many other diseases (14).

## Sialic acid and atherosclerosis

### Chemical modification of lipoproteins and its impact

The main protein components of LDL, namely apolipoprotein B-100 and GM2 gangliosides, are sialylated. For example, the main LDL APOB100 protein contains about a dozen sialic acid residues covalently bound to the ends of its N-linked glycans. Their removal may alter the biological and chemical properties of APOB100. Intermediate-density lipoproteins (IDL) and very low-density lipoproteins (VLDL) become more susceptible to self-association (15). Stimulation of cholesterol accumulation in the smooth muscle cells of the human aortic intima occurs due to the desialylation of APOB100. It has also been confirmed that it promotes the absorption of cholesterol by macrophages. It can be argued that atherogenic (i.e., desialylated) LDL is smaller and has a higher density. It also contains fewer neutral carbohydrates, essential lipids, vitamins, and antioxidants. Another distinguishing factor is that desialylated LDL is more susceptible to copper-dependent oxidation and self-association (16,17).

Other modifications that LDL can undergo *in vivo* are glucosylation, glycol oxidation, oxidation, and deglycosylation, which also make it atherogenic. In addition, the glucosylation of desialylated LDL increases its atherogenic potential. Thus, due to its qualities, glycosylation can be considered an additional risk factor for the development of atherosclerosis in people with diabetes mellitus (18). The atherogenicity of LDL does not increase either by degalactosylation of already desialylated LDL (i.e., cleavage of galactose (Gal) covalently bound to the terminal Sia) or by its complete deglycosylation (i.e., elimination of carbohydrates). Thus, we can assume that desialylation plays a crucial role in the development of LDL atherogenicity (19).

It is widely known that high cholesterol (associated with LDL) is one of the main risk factors for heart disease. In addition, people with normal LDL levels have about 50% of initial cardiovascular events. That is why it is widely believed that atherosclerosis is not caused by an increase in the level of LDL in plasma but by changes in the composition of LDL. Thus, in 1989, plasma LDL was first reported to have higher atherogenicity in people with cardiovascular diseases (20). It was also found that LDL sialidase treatment significantly contributed to an increase in cholesterol deposition in cultured human aortic intima cells and to the transformation of macrophages, endothelial cells, and artery cells (primarily pericytes) into foam cells. In addition, the level of sialic acid in LDL particles was found to be several times lower in people with coronary heart disease (CHD). Based on these results, it was suggested that desialylation of LDL induces atherogenicity (21).

### Trans-sialidases in atherosclerosis

The concentration of trans-sialidase in blood serum ranges from 20 to 200 mcg/mL. The molecular weight of this enzyme is 65 kDa, and it has three optimum pHs: 3.0, 5.0, and 7.0. It can act in the blood and cellular organelles with a lower pH (for example, inside the lysosome) (22). Trans-sialidase can cleave sialic acids from LDL, VLDL, IDL, and high-density lipoproteins (HDL). It also transfers them to various receptors in human plasma. Treatment of native LDLs with purified trans-sialidase leads to the desialylation of LDL. This is the reason for the accumulation of cholesterol esters in smooth muscle cells of the human aorta intima *in vitro*. Thus, it can be assumed that trans-sialidase affects the formation of foam cells (23).

Golovanova et al. (24) determined the difference in the activity and specificity of sialidases in atherosclerotic lesions compared to unaffected intima. The authors compared the activity of sialidases in homogenates of human aortic intima by measuring the amount of ganglioside-monosialic acid (GM1) formed during the incubation of ganglioside GD1a with tissue homogenates. For comparison, areas with and without atherosclerotic lesions were taken separately.

One of the pathological pathways involved in the triggering of atherogenesis in the human body comprises the transmission of toll-like receptor (TLR) signals. TLRs can be considered a link between the immune system and cardiovascular diseases. Due to the activation of these receptors, many intracellular signal transmission pathways are triggered, which leads to the production of pro-inflammatory cytokines (25). TLR is known to stimulate the synthesis of interleukin-1 beta (IL-1β). It was also found that both TLR and IL-1β are involved in the development of atherosclerosis (26,27).

The physiological role of trans-sialidase in human blood plasma remains unknown. The enzyme participates in processes that depend on the sialylation and desialylation of various cellular and non-cellular components. By changing the lifespan of glycoproteins, lipoproteins, and cells, as well as influencing intercellular interactions, trans-sialidase can modulate the activity of plasma enzymes. At the same time, trans-sialidase (as a factor of atherogenic LDL change) can play a decisive role in atherogenesis (28). Due to its properties, trans-sialidase can influence the interaction of lipoproteins and arterial cells. Intracellular lipid accumulation is stimulated by LDL desialylated by trans-sialidase, followed by stimulation of proliferative activity and synthesis of extracellular matrix. Thus, desialylation of LDL by trans-sialidase causes almost all known cellular manifestations of atherosclerosis. In one study, it was found that the activity of sialidase in the blood serum of patients with CVD was increased. The authors did not indicate which of the enzymes affected the activity of sialidase. In addition, the content of sialic acid in LDL correlated with atherosclerosis (29).

An increase in the activity of neuraminidase in blood serum, together with a decrease in the content of sialic acid in LDL, led to a stabilization of free sialic acid concentration in animal models. Probably, the absence of an increase in sialic acid plasma concentration may be due to the activity of trans-sialidase, which transfers sialic acid residues from LDL to other plasma glycoconjugates (30).

In one study conducted on a rabbit model, it was revealed that the binding of fibrinogen and LDL to the surface of the artery wall was weakened by sialic acid located on the surface. At the same time, removal of sialic acids from the surface of artery walls by treatment with sialidase led to the formation of neointima. This was due to the proliferation of smooth muscle cells in the walls of blood vessels. Based on this, it can be said that sialic acids inhibit the proliferation of smooth muscle cells and intima thickening (31).

Circulating immune complexes consisting of LDL and autoantibodies to LDL were found in the blood of most patients with coronary atherosclerosis. It was found that the development of atherosclerosis is directly related to the number of circulating immune complexes that contain LDL (32,33). It is important to note that LDL in circulating immune complexes is identical to the desialylated LDL subfraction. Autoantibodies of immune complexes are IgG, which interacts with the protein and not with the lipid part of LDL. The accumulation of lipids in smooth muscle actin-positive (SMA (+)) cells cultured from the intact intima of the human aorta is facilitated by complexes of autoantibodies with native LDL. Autoantibodies increase the atherogenic potential of desialylated LDL due to the formation of complexes with lipoproteins. At the same time, it was found that IgG molecules exhibit sialidase activity in people with coronary sclerosis. It should be clarified whether IgG can also have sialidase activity in this case (34).

### Sialidases in atherosclerosis

It has been proven that due to its influence on inflammatory reactions, insulin resistance, and lipid metabolism, NEU1 can participate in the development of coronary atherosclerosis. In their study, Yang et al. (35) found lower levels of non-HDL cholesterol in hypomorphic NEU1 mice, which had reduced NEU1 activity. Due to a decrease in endocytosis through the ASGPR (asialoglycoprotein receptors) of human hepatocytes, hypomorphic expression of NEU1 led to an increase in LDLR (LDL receptor) stability. In addition, a reduced level of proprotein convertase subtilisin/kexin 9 (PCSK9), which can bind to LDLR and target its degradation, was detected in hypomorphic NEU mice. Also, a higher level of cholesterol in the liver caused a decrease in the content of the protein that binds the steroid response element-2 (SREBP-2), which is mandatory for the promoter of the microsomal triglyceride transporter (necessary for the synthesis of VLDL) (36,37). Consequently, lower VLDL production in the liver and higher LDL uptake were observed. Due to this, there was a decrease in the risk of CAD. In other studies, it was found that compared with Apoe−/− mice, NEU1 hypomorphic mice show a significantly reduced size of atherosclerotic lesions (a decrease of >50%) (38). Also, in hypomorphic NEU1 mice, fewer T cells, macrophages, and smooth muscle cells were found in the aortic root. This indicates a lower degree of inflammation and recruitment of cells inside the plaque. Thus, downregulation of NEU1 can decrease atherosclerosis, lower cholesterol (other than LDL), and inhibit the transmigration of leukocytes in mice with Apoe knockout (39,40).

Neu3 is involved in cell growth, differentiation, and migration. There is also evidence that this sialidase may be involved in the thickening of intima. In vascular smooth muscle cells, overexpression of Neu3 inhibited the expression of matrix metalloproteinase-9 (MMP-9). This enzyme can be a physiological modulator of vascular smooth muscle cell reactions and can also participate in the destabilization of plaques in atherosclerosis (41).

Sialidases can affect the activity of many lipoproteins and blood cells. During experiments on C57Bl/6 mice, changes occurred in lipoprotein metabolism due to the expression of hypomorphic sialidase. Also, this expression was sufficient to weaken atherogenesis, as there was an anti-atherosclerotic effect when apoE-/- mice were injected with 2-deoxy-2,3-dehydro-N-acetylneuramic acid (DANA) (42). In addition, it was found that by increasing the absorption of cholesterol by monocytes and the outflow of cholesterol by macrophages into HDL, hypomorphic expression of sialidase provides atheroprotection. Taken together, these observations suggest that hypomorphic expression of sialidase is atheroprotective in C57Bl/6, ApoE-deficient, and LDLR-deficient mouse models. Because sialidases may be a new risk factor for atherosclerosis, it becomes especially important to further study the contribution of genetic variations in sialidase genes to the development of atherosclerotic cardiovascular diseases in humans (43).

### Sialyltransferases in atherosclerosis

ST were found to be involved in the re-synthesis of sialic acid on the surface of endothelial cells after injury. During CAD, ST activity in the intima and blood plasma was increased. The sialic acid level on the cell surface *in vitro* immediately decreased when the endothelial cell was damaged (44). After 24 h, it increased, which corresponded to an increased content of ST. There is an assumption that the increased activity of ST in atherosclerotic intima and plasma is a self-defense mechanism to accelerate the restoration of the surface of endothelial cells and, accordingly, delay the development of atherosclerosis after injury. At the same time, due to the increased chemotaxis of inflammatory cells, overexpression of ST contributed to the formation of atherosclerosis (45,46). The CXC chemokine receptor 2 (CXCR2) is involved in leukocyte adhesion, which is caused by chemokine action. It can also take part in the migration of mononuclear cells to atherosclerotic lesions. Due to the sialylation of CXCR2, ST3Gal-IV significantly accelerated the CXCR2-mediated adhesion of leukocytes and the transformation of macrophages into foam cells. ST3Gal-IV knockout contributed to a decrease in the spectrum of atherosclerotic lesions and the content of inflammatory cells in the atherosclerotic plaque in Apoe−/− mice that received a high-fat diet (47,48).

Thus, overexpression of ST contributes to the restoration of endothelial cells in the arteries. But, at the same time, due to its influence on recruitment, ST also accelerates the formation of atherosclerosis.

### Sia-siglecs axis of immune cells

Considering inflammation, the Sia (sialic acid)-siglecs (sialic acid-binding immunoglobulin-like lectin) axis is of special importance. Siglecs are negative regulators of the wide range of immune cells, which act as “self” sensors to protect against the overreaction of the immune system (49). Not all functions and metabolism details of Siglecs are clear, but they are known to be essential immuno-modulatory receptors that alter immune cell function by binding to Sia. The balance within the Sia-Siglec axis corresponds to the balance between immune response and immune tolerance. The majority of siglecs have an intracellular immunoreceptor tyrosine-based inhibition motif that mediates inhibitory signals. Sia-siglecs binding can occur as cis- (siglecs bind to sia exposed on the same cell) and trans- (cell expressing siglecs binds to sia or glycoproteins present on another cell) interactions (50).

Siglec-G, which is mostly expressed in B-1 cells, was demonstrated to stimulate liver inflammation and atherosclerosis via suppression of B-1 cells' protective function (51). In LDLR-/- mice with Siglec-G deficiency, an enhanced level of B-1 cell-derived IgM was observed, which exceeded physiological plasma levels. IgM appeared to suppress the proinflammatory properties of OxLDL, preventing foam cell formation and the accumulation of apoptotic cells by accelerating their uptake by macrophages (52).

Dendritic cells (DC), as a part of the group of antigen-presenting cells, are important components of immune system balance. The immunogenicity of sialic acid-modified antigens is changed. During inflammatory response, the sialylated antigen inhibited immunization by binding to Siglec-E of DCs in an antigen-specific tolerogenic manner (53). In particular, the number of Treg cells was increased, while the IFN-γ production, as well as the expansion of effector T cells, was suppressed. Tregs were shown to have atheroprotective properties, which is supported by the finding of decreased number of cells of this type in patients with coronary atherosclerosis (54). According to this, sialylation of specific antigens can be a promising target to improve atherosclerosis by modulating the effects of DC cells on Treg cells and effector T cells.

Siglec-1 (sialoadhesin, CD169) is expressed on macrophages within the plaque of atherosclerotic patients and on circulating monocytes. Siglec-1-positive macrophages participate in the process of endothelial cell adhesion, lipid internalization, antigen presentation, and pro-inflammatory cytokines secretion. In Apoe-/- mice, blockage of Siglec-1 by lentivirus-mediated siRNA appeared to potentially prevent atherosclerotic lesion formation and proinflammatory cytokine production. Moreover, Siglec-1 is a potential risk marker for CAD and a potential non-invasive indicator for monitoring disease severity (55). Upregulated Siglec-1 expression on monocytes was linked to enhanced T-cell proliferation in CAD patients (56). On the other hand, down-regulation of Siglec-1 may diminish the proliferation and activation of cocultured T effector cells (56). Thus, Siglec-1 is an important contributor to the inflammatory aspect of atherosclerosis, the impact of which is implemented via the secretion of chemokines and pro-inflammatory cytokines.

## Therapeutic implications

Sialic acids and sialidases are incorporated in atherosclerosis pathogenesis in numerous ways, which makes them promising targets for therapeutic strategies. Chemical modifications of lipoprotein (LP) can be useful in the diagnosis of cardiovascular diseases, evaluation of available treatment options, and cure of the diseases themselves. There are several methods of screening panels of experimental drugs for the presence of compounds with anti-atherogenic activity. It is also possible to evaluate the activity of trans-sialidase and sialidase in plasma and quantify modified LDL, LDL-CIC (low-density lipoprotein circulating immune complex), and anti-LDL antibodies (57).

Neu5Ac is another promising target. Oseltamivir and zanamivir, which are usually used for influenza treatment, have shown an ability to inhibit NEU1 and thus decrease Neu5Ac (58).

The available data indicate that transfusion of lipoprotein particles containing plasma enzymes, antiatherogenic plasma lipids, and apolipoproteins can be used in new therapeutic approaches. At the moment, several examples of successful antiatherogenic therapy using LCAT (lecithin:cholesterol acyltransferase) and HDL enriched with native ApoAI are known. At the same time, lipoprotein particles intended for transfusion can be enriched with antioxidants, vitamins, and anti-atherogenic lipids (such as phosphatidylcholine isomer 34:2, palmitoillinoleoyl phosphatidylcholine). It has been also demonstrated that with the help of immobilized LDL and LDL apheresis, non-lipid atherogenicity factors (antibodies against LDL) can be removed from the blood. Also, a specific sialidase inhibitor that reduces lipid uptake by artery cells and increases cholesterol outflow to HDL and LP sialylation may be used (57).

All these observations again demonstrate the potential of sialic acid and its metabolism as a therapeutic target. In addition, the variety of mechanisms through which sialic acids participate in atherogenesis leads to variability in the therapies that can be beneficially used.

## Conclusions

Sialic acids can participate in many cellular events and the overall immune response, and therefore their involvement in atherosclerosis processes is quite logical. LDL desialylation plays a significant role in the initiation and development of the pathological process of atherosclerosis. According to modern concepts, the multiple modifications of LDL particles are the main trigger and one of the main events of atherogenesis. Among these modifications, oxidation, glycosylation, and desialylation occupy a special place. Low content of sialic acids in LDL is associated with increased cholesterol accumulation in human aortic smooth muscle cells and macrophages. Since the accumulation of cholesterol esters in the intima of the arteries is one of the earliest manifestations of atherosclerosis, its development may be affected by a change in the level of sialic acids in LDL.

Another mechanism through which sialic acids impact atherogenesis is inflammation. A promising subject for further research is the sia-siglecs axis of immune cells, which is implicated in the process of atherosclerosis progression.

Multiple studies strongly suggest that sialic acids are a promising target for developing therapeutic strategies.
